# Effects of Evolocumab Added to Moderate-Intensity Statin Therapy in Chinese Patients With Acute Coronary Syndrome: The EMSIACS Trial Study Protocol

**DOI:** 10.3389/fphys.2021.750872

**Published:** 2021-11-23

**Authors:** Jing Gao, Jing-Yu Liu, Peng-Ju Lu, Jian-Yong Xiao, Ming-Dong Gao, Chang-Ping Li, Zhuang Cui, Yin Liu

**Affiliations:** ^1^Thoracic Clinical College, Tianjin Medical University, Tianjin, China; ^2^Cardiovascular Institute, Tianjin Chest Hospital, Tianjin, China; ^3^Department of Cardiology, Tianjin Chest Hospital, Tianjin, China; ^4^School of Public Health, Tianjin Medical University, Tianjin, China

**Keywords:** evolocumab, acute coronary syndrome, low-density lipoprotein cholesterol, adverse cardiovascular events, proprotein convertase subtilisin/kexin type 9

## Abstract

**Background:** Several studies have demonstrated that using a higher dose of statin can easily induce liver injury and myopathy. Low-density lipoprotein cholesterol (LDL-C) is a well-established modifiable risk factor for cardiovascular disease; however, the large majority of Chinese patients cannot meet the target level of LDL-C recommended by the Chinese expert consensus. Evolocumab has been demonstrated to reduce LDL-C by approximately 60% in many studies. Nevertheless, whether combined evolocumab and moderate-intensity statin is as effective in lowering LDL-C and decreasing incidence of MACE in Chinese patients presenting with the acute phase of acute coronary syndrome (ACS) remains unknown. Therefore, the “Evolocumab added to Moderate-Intensity Statin therapy on LDL-C lowering and cardiovascular adverse events in patients with Acute Coronary Syndrome” (EMSIACS) is conducted.

**Methods:** The EMSIACS is a prospective, randomized, open-label, parallel-group, multicenter study involving analyzing the feasibility and efficacy of evolocumab added to moderate-intensity statin therapy on lowering LDL-C levels in adult Chinese patients hospitalized for acute phase ACS. The sample size calculation is based on the primary outcome, and 500 patients will be planned to recruit. Patients are randomized in evolocumab arm (evolocumab 140mg every 2weeks plus rosuvastatin 10mg/day or atorvastatin 20mg/day) and statin-only arm (rosuvastatin 10mg/day or atorvastatin 20mg/day). The primary outcome is the percentage change in LDL-C in weeks 4 and week 12 after treatment. The secondary outcome is the occurrence of MACE after 12weeks and 1year of treatment.

**Discussion:** If the EMSIACS trial endpoints prove statistically significant, the evolocumab added to moderate-intensity statin therapy will have the potential to effectively lower subjects’ LDL-C levels, especially for the Chinese patients with acute phase ACS. However, if the risk of MACE is not significantly different between the two groups, we may extend follow-up time for secondary outcome when the clinical trial is over.

**Clinical trial registration**: The study is registered to ClinicalTrials.gov (NCT04100434), which retrospectively registered on November 24, 2020.

## Introduction

In recent years, acute coronary syndrome (ACS) has been among the leading causes of cardiovascular death in China (Huo et al., 2017). It is characterized by fast onset, rapid development, poor prognosis, and high mortality (Gao et al., 2018). Abnormal lipid metabolism is one of the most significant risk factors of ACS. Elevated low-density lipoprotein cholesterol (LDL-C) is highly associated with adverse events such as cardiovascular disease, coronary artery disease, and major adverse cardiovascular events (MACE). Lowering LDL-C will reduce the risk of cardiovascular events (Sabatine et al., 2018). In recent years, LDL-C lipid-lowering therapy for the treatment of ACS and coronary heart disease (CHD) has been focused. Gao et al. (2014) systematically analyzed the effects of LDL-C lipid-lowering therapies on changes in the volume of coronary atherosclerotic plaque in patients with coronary heart disease and conducted evidence-based medical research supporting the application of LDL-C lowering therapies for clinical plaque treatment. Chinwong et al. (2015) suggested that achieving target LDL-C levels of <70mg/dl (<1.8mmol/L) in patients with ACS could significantly reduce the risk of cardiovascular events.

Expert consensus or guidelines have recommended treatment goals of LDL-C level. The 2019 ESC/EAS Guidelines for the management of dyslipidemias (Mach et al., 2019) stated that for patients at high risk, an LDL-C reduction of ≥50% from baseline and an LDL-C goal of <1.8mmol/L (<70mg/dl) are recommended; for patients at very-high risk, an LDL-C reduction of ≥50% from baseline and an LDL-C goal of <1.4mmol/L (<55mg/dl) are recommended. For the Chinese population, similar goal is proposed. According to the 2020 Chinese expert consensus on lipid management of very high-risk atherosclerotic cardiovascular disease patients (ASCVD patients; Atherosclerosis and Coronary Heart Disease Working Group of Chinese Society of Cardiology and Editorial Board of Chinese Journal of Cardiology, 2020), LDL-C level should be lowered to <55mg/dl (1.4mmol/L) AND reduction of LDL-C is ≥50% from baseline. For patients at very high-risk (defined as either having ≥2 serious ASCVD events or 1 serious ASCVD event combined with ≥2 high-risk factors), the double compliance of LDL-C lowering targets is required for achieving success in treatment for the Chinese population.

Statins have been widely used as an effective LDL-C lowering agent for ASCVD patients (Wang et al., 2017; Fox et al., 2018). However, many patients are unable to achieve the recommended LDL-C goal after high-intensity statin therapy (Wang and Fang, 2018). In addition, statins have the disadvantage of delayed onset of action (about 2weeks; Kakara et al., 2014; Koskinas et al., 2018). Genetic polymorphism associated with statin-induced myopathy in Chinese coronary artery disease patients (Liu et al., 2017). Several studies have demonstrated that using a higher dose of statin can easily induce liver injury (Alsheikh-Ali et al., 2007; Kasliwal et al., 2007; Chen et al., 2014; Naiqiong et al., 2017; Bandyopadhyay et al., 2018).

To achieve the target reduction in LDL-C, combinatory therapy using non-statin agents added to statin has been developed. Several recent trials have shown that the addition of either ezetimibe or anti-proprotein convertase subtilisin/kexin type 9 (PCSK9) monoclonal antibodies to statin therapy provides a further reduction in ASCVD risk, which is positively correlated with the incrementally achieved absolute LDL-C reduction (Giugliano et al., 2017, 2018; Schwartz et al., 2018; Koskinas et al., 2019). Current guidelines or expert advice have further recommended using PCSK9 inhibitors for high-risk or very high-risk patients given that the ezetimibe added to statin therapy failed to achieve the target LDL-C level. In the 2019 ESC/EAS Guidelines for the management of dyslipidemias (Mach et al., 2019), it is stated that for secondary prevention, patients at very high risk not achieving their goal on a maximum tolerated dose of statin and ezetimibe, a combination with a PCSK9 inhibitor is recommended. In the China cholesterol education program (CCEP) expert advice for the management of dyslipidemias to reduce cardiovascular risk (2019; China Cholesterol Education Program Working Committee et al., 2020), it is recommended to add PCSK9 inhibitors if the LDL-C goal of <55mg/dl (<1.4mmol/L) is not achieved after 4–6weeks of statin combined with ezetimibe therapy. In addition, for patients who may fail to achieve LDL-C<55mg/dl (<1.4mmol/L) with the ezetimibe added to statins therapy, PCSK9 inhibitors added to statins can be applied directly.

Evolocumab is a fully human monoclonal antibody against PCSK9. To date, several studies have been investigating the efficacy of evolocumab added to statin therapy in reducing the risk of cardiovascular events (Sabatine et al., 2017). It has been well demonstrated that evolocumab can effectively diminish dyslipidemia under various design settings (Raal et al., 2015; Koskinas et al., 2019). Nevertheless, whether the “evolocumab added to moderate-intensity statin” therapy is as effective in lowering LDL-C level in Chinese patients with acute phase ACS (i.e., the risk of recurrence of the event was extremely high within the first month of initial disease onset) remains to be elucidated. In addition, as statins may have side effects of liver damage, muscle pain, and increased risk of type 2 diabetes (Bandyopadhyay et al., 2018) and have favorable properties including anti-inflammation, platelet inhibition, and plaque stabilizing effects (Antonopoulos et al., 2012; Sikora et al., 2013; Camargo et al., 2014), comprehensive physical examination and laboratory blood tests including fasting glucose level, biomarkers of inflammation, liver and kidney functions panel, and platelet reactivity should be evaluated. Exploring these mechanistic effects can assist in advanced understanding of the clinical benefits of the present evolocumab on top of statin therapy.

### Aims of the Study

The overall purpose of EMSIACS study is to determine the feasibility, safety, and efficacy of the evolocumab added to moderate-intensity statin therapy, using the statin-only therapy as the control arm, on LDL-C level variability between baseline and 1-year follow-up in patients with acute phase ACS. This study was designed to address two principal research questions:

1. We determine the effects of the evolocumab added to moderate-intensity statin therapy on MACE.
2. We plan to identify the appropriate ACS patient population for the administration of evolocumab in combination with standard statin therapy.

## Materials and Methods

### Patient and Public Involvement

This is a prospective, open-label, randomized, parallel-group, and multicenter study. We are planning to include 500 patients with acute phase ACS (non-ST-segment elevation myocardial infarction (MI), acute ST-segment elevation MI within 24h of onset, and unstable angina within 72h of onset). Patients will be randomly assigned in a 1:1 ratio using a computer-generated random number system (block randomization with blocks of size 4 at random) and are divided into two treatment groups: statin-only therapy and evolocumab plus statin therapy. A 48-week follow-up study will be conducted.

### Design and Setting

This trial has been registered in ClinicalTrials.gov (No. NCT04100434). The dates of the study estimated from January 1, 2021, to June 1, 2023.

This study will be conducted in the Tianjin Chest Hospital, China. Patients will be recruited from eight grade III Level A hospitals across the Tianjin area. The comprehensive analysis of baseline demographic characteristics, clinical examination, laboratory tests, and investigation of the forecast value will be conducted in the Tianjin Chest Hospital.

### Ethical Considerations

The study protocol has been reviewed and approved by the Ethics Committee (IEC) of the Tianjin Chest Hospital, China (No. 2019KY-019-01).

### Drug Usage and Dosage

**Figure 1:**
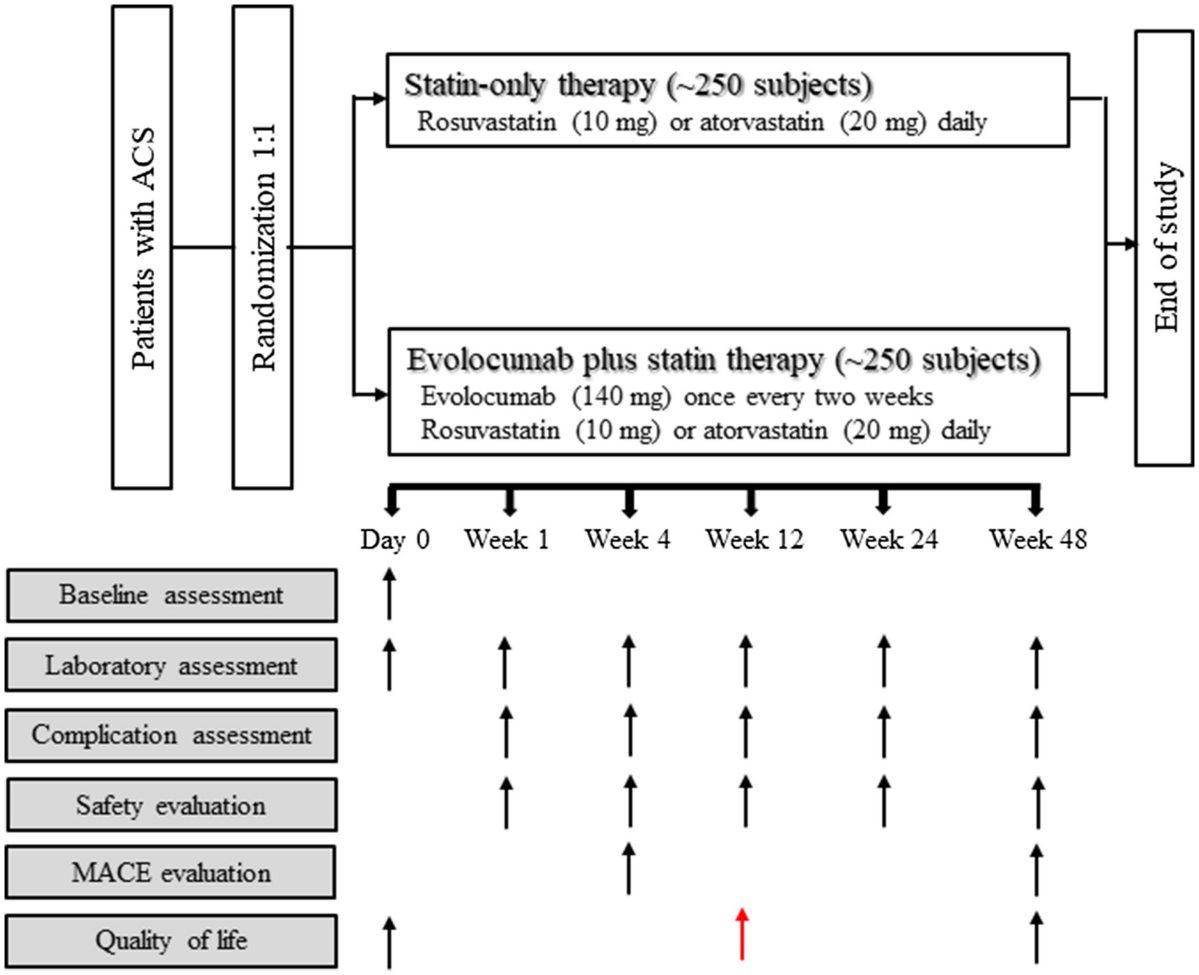
Study flowchart. Reasons for inclusion and exclusion of patients are detailed in Materials and Methods section. ACS, acute coronary syndrome; MACE, major adverse cardiac event.

The usage and dosage of evolocumab and statins used in the present study are as follows (Figure 1):

1. Statins: according to the recommendations of the 2020 edition of the Expert Consensus on Clinical Pathway of Blood Lipid Management in Patients with Acute Coronary Syndrome (Chinese Medical Doctor Association and Expert Group of Clinical Pathway of Lipid Management in Patients With Acute Coronary Syndrome, 2020), all patients with ACS are treated with moderate-intensity statins, rosuvastatin (10mg) or atorvastatin (20mg) daily throughout the study period.
2. Evolocumab (trade name: Repatha®): patients will be given evolocumab within 72h immediately after diagnosis of ACS, with subcutaneous administration of 140mg evolocumab once every 2weeks (total 24 time points during the 1-year follow-up period). Use a single-use prefilled auto-injector to administer evolocumab subcutaneously in areas of the abdomen, thighs, or upper arms that are not tender, bruised, red or hardened. Within 30min, three injections will be given continuously with a single-use prefilled auto injector.

### Subject Selection – Inclusion and Exclusion Criteria

Patients admitted to one of the eight participating hospitals with acute ACS are selected, and the recruitment process is as follows: (1) for patients who are going to receive the lipid-lowering therapy, assess whether they are suitable for the trial according to the inclusion and exclusion criteria; (2) for patients who fulfill the trial criteria, introduce and explain the study; patients will be enrolled only after they agree to participate and provide signed informed consent; (3) for patients who refuse to sign the informed consent form, withdraw them from this trial, and perform conventional clinical practice.

Patients aged ≥18 and ≤85 with recent hospitalization for acute phase ACS are eligible for inclusion in this study. The diagnosis for ACS in this study is defined as non-ST-segment elevation myocardial infarction (MI) within 24h of onset, acute ST-segment elevation MI within 24h of onset, or unstable angina within 72h of onset. Additional inclusion criteria are based on patients with elevated LDL-C levels who meet one of the following three conditions: (1) prior to the study, patients who received intensive statins for more than 4weeks (the same dose of statin therapy has been sustained for the past 4weeks), having LDL-C levels ≥70mg/dl (≥1.8mmol/L) or non-HDL-C≥100mg/dl (≥2.6mmol/L); (2) prior to the study, patients who received moderate-intensity statin therapy for more than 4weeks (the same dose of statin therapy has been sustained for the past 4weeks), having LDL-C levels ≥90mg/dl (≥2.3mmol/L) or non-HDL-C≥120mg/dl (≥3.1mmol/L); (3) prior to the study, patients who do not receive any statin therapy or discontinue statin therapy, having LDL-C≥125mg/dl (≥3.2mmol/L) or non-HDL-C≥155mg/dl (≥4.0mmol/L); and (4) prior to the study, patients who do not receive any statin therapy or discontinue statin therapy, having LDL-C L125 mg/dL (≤3.2mmol/L).

Patients with the following conditions are excluded: (1) patients unable to understand the research requirements or refuse to sign the informed consent form; (2) patients with unstable clinical status (e.g., hemodynamics or ECG instability); (3) patients with uncontrolled arrhythmia, defined as recurrent or symptomatic ventricular tachycardia and atrial fibrillation with rapid ventricular reaction that the drug cannot control within 3months prior to screening; (4) patients with severe renal insufficiency, defined as the estimated glomerular filtration rate (eGFR)<30ml/min/1.73m^2^; (5) patients with active liver diseases or liver dysfunction, either recorded in the patient’s medical record or defined as an increase in aspartate aminotransferase (AST) or alanine aminotransferase (ALT) more than 3 times above the upper limit of normal range; (6) patients with intolerance to rosuvastatin (any dose) or to other statins; (7) patients with known allergies to contrast agents, heparin, aspirin, ticagrelor, or clopidogrel; (8) patients with known allergies to supplements required for the use of the drug; (9) patients who have been treated with evolocumab or other PCSK9 inhibitors; (10) patients who have received cholesterol ester transfer protein inhibitors treatment 12months prior to screening; (11) patients who have received systemic steroid or cyclosporine treatment in the past 3months; (12) patients with known infections, hemorrhages, metabolic or endocrine disorders as determined by the researchers; (13) patients who have been included in other studies; (14) patients with active malignant tumor in need of treatment; and (15) women who are fertile (age<50years, menstruation in the past 12months) and have not received tubal ligation, oophorectomy, or hysterectomy.

### Data Collection and Management

Data will be recorded in Case Report Forms (CRFs) and entered into the clinical trial center by an electronic data capture system. Baseline data and data of different follow-up time points will be recorded. The schedule for the baseline assessment and subsequent assessments at different follow-up time points is shown in Table 1. Researchers on the trial management team will ensure the completeness and accuracy of the data. The trial manager, principal investigator, statisticians, and other members of the trial team will review the data cooperatively and complete the final definition and judgment of population analysis. The database will be secured.

**Table 1:** Schedule of treatments and assessments administered to patients.

Research stage	Screening stage	Follow-up treatment stage															
Visit (V)	V1	V2			V3	V4	V5	V6	V7	V8	V9	V10	V11	V12	V13	V14	V15
Date (D)	D-3 to D0	D1	D2	D3	D7	D14	W4 ± 3	W6 ± 3	W8 ± 3	W10 ± 3	W12 ± 3	W16 ± 3	W20 ± 3	W24 ± 3	W32 ± 3	W40 ± 3	W48 ± 3
Visit window																	
Inclusion/exclusion criteria	✓	✓															
Informed consent	✓																
Medial history	✓																
^*^Evolocumab treatment	✓																
Physical examination^(1)^	✓																
Vital signs^(2)^	✓	✓															
Blood routine^(3)^	✓				✓		✓				✓			✓			✓
Cardiac enzymes^(4)^	✓				✓		✓				✓			✓			✓
Blood lipids^(5)^	✓				✓		✓				✓			✓			✓
Blood sugar	✓				✓		✓				✓			✓			✓
hemoglobin A1c	✓						✓				✓			✓			✓
Liver function and renal function^(6)^	✓				✓		✓				✓			✓			✓
D-dimer	✓				✓		✓				✓			✓			✓
Uric acid	✓				✓		✓				✓			✓			✓
NT-proBNP	✓				✓		✓				✓			✓			✓
Thyroid function^(7)^	✓						✓				✓			✓			✓
High-sensitivity C-reactive protein	✓				✓		✓				✓			✓			✓
Ultrasound cardiogram	✓						✓				✓			✓			✓
PROMIS 10	✓										✓						✓

* Evolocumab is administered once every 2weeks.

(1) Physical examination: height and weight.

(2) Vital signs: body temperature, blood pressure and heart rate.

(3) Blood routine: RBC, WBC, platelet, and hemoglobin.

(4) Cardiac enzymes: creatine kinase (CK), CK-MB, troponin T (TnT), and Troponin (TnI)

(5) Blood lipids: low-density lipoprotein cholesterol (LDL-C), high-density lipoprotein cholesterol (HDL-C), very low-density lipoprotein cholesterol (VLDL-C), total cholesterol, triglycerides, apolipoprotein B, apolipoprotein A-1, lipoprotein (a), and free fatty acids.

(6) Liver function and renal function: aspartate aminotransferase (AST), alanine aminotransferase (ALT), alkaline phosphatase (ALP), γ-glutamyltransferase (γ-GT), acetylcholinesterase, mitochondrial isoenzymes, total bilirubin, creatine, and estimated glomerular filtration rate (eGFR)

(7) Thyroid function: thyroid-stimulating hormone (TSH), thyroid hormones, and triiodothyronine.

### Baseline Assessments and Laboratory Examinations

At baseline, the following characteristics are assessed: (1) demographic characteristics (gender, date of birth), (2) physical status (height, weight, body temperature, blood pressure, heart rate), (3) medical history (hypertension, diabetes, hyperlipidemia, heart disease history, peptic ulcer, stroke history), (4) treatment history (coronary heart disease, names of drugs used), (5) family history (hypertension, diabetes, coronary heart disease, hyperlipidemia, stroke), (6) life style (smoking, drinking), (7) current medical history, (8) echocardiography (left atrium, left ventricle, left ventricular ejection fraction, pulmonary artery pressure), and (9) laboratory examinations.

The laboratory examinations will be performed at baseline and at weeks 1, 4, 12, 24 and 48 post-treatment and will include the following: routine blood tests [blood glucose, liver function panel [albumin, total and direct bilirubin, alkaline phosphatase, aspartate aminotransferase, alanine aminotransferase], kidney function panel (serum creatinine, blood urea nitrogen)]; serum lipids panel [LDL-C, non-HDL-C, total cholesterol, triglycerides, apolipoprotein B, apolipoprotein A1, lipoprotein(a)]; myocardial damage markers [serum creatine kinase (CK), CK-MB, troponin I (TnI), and troponin T (TnT)]; and quality of life assessment using the PROMIS 10 scale (a Global Physical Health Score and a Global Mental Health score; Cella et al., 2007).

### Treatment Procedures Records

Treatment-related procedures will be recorded, including all medication used; percutaneous coronary intervention (PCI), coronary artery bypass (CABG) or thrombolytic therapy for revascularization, coronary angiography of the diseased vessels (e.g., left anterior descending artery, left circumflex artery, left main artery, right coronary artery, tunneled artery), stent implantation factors (target vessels for stent implantation, number of stents, stent diameter, stent length, stent overlapping); and postoperative lesions (degree of residual diameter stenosis, thrombolysis in myocardial infarction (TIMI) grade flow).

### Main Outcome Measures

Subjects of the present study will receive 24 treatments after being enrolled, and data of laboratory examinations will be evaluated at 72h and weeks 1, 4, 8, 12, 24, and 48 after intervention (Table 1; Figure 1).

The primary outcome of the present study will be the mean percentage change of the LDL-C level between baseline and weeks 4 and 12 post-treatment (Table 1; Figure 1). The secondary outcome will be MACE (defined as coronary heart disease death, nonfatal myocardial infarction, hospitalization for unstable angina, unplanned coronary revascularization, and stroke) assessed at week 12 and week 48 post-treatment (Table 1; Figure 1). The following issues will also be investigated: (1) the proportion of patients with LDL-C<70mg/dl, <55mg/dl and<40mg/dl during the study period; (2) patients’ quality of life as evaluated with the PROMIS 10 scale at week 12 and week 48 post-treatment; and (3) the percentage change in inflammatory biomarkers including but not limited to high-sensitivity C-reactive protein (hsCRP), interleukin (IL)-1β and IL-6, from baseline to weeks 48 (Table 1; Figure 1). Other biomarkers that exhibit significant difference between the two study arms will be investigated retrospectively after the trial.

### Safety Evaluation

For safety outcome analysis, missing data will not be imputed. Adverse events (AEs) will be recorded and summarized using descriptive statistics. Tolerability of study drug will be monitored and evaluated throughout the study period *via* regular medical examination and laboratory tests. For analyses of clinical endpoints, incidence of AEs and/or serious adverse events (SAEs) will be summarized for each randomized treatment group. Serious adverse events include cardiovascular events (death, fatal or non-fatal MI, coronary revascularization, hospitalization for recurrent ACS, hospitalization for heart failure, stroke, transient ischemic attack), muscle-related adverse events, neurocognitive impairments, incident diabetes; adverse events of special interest include allergies, injection-site reactions, or any other clinical abnormalities. If unexpected or SAE occur, the researchers or staff will fill out the SAE Report Form and notify the Ethics Committee and the State Food and Drug Administration (SFDA) as well as the pharmacological authorities.

Possible intervention-related complications will be recorded at 72h, and weeks 1, 4, 8, 12, 24, and 48 after intervention, including: dissection, acute thrombosis, coronary perforation, puncture site complications, allergic reactions, vasovagal response, perioperative myocardial infarction, cerebrovascular events, and hypotension.

### Study Termination

Patients have the right to withdraw from the proposed study at any time without reason. Such withdrawal will not affect the patients’ rights to receive appropriate medical management in the future. However, researchers will terminate the study for patients having the following situations: poor adherence to the treatments; serious adverse drug reactions; pregnancy; patients refusing to continue participating in the study; lost to follow-up; or other safety reasons.

Excessively high withdrawal rates may lead to unexplained results. Therefore, unnecessary withdrawal should be avoided. Withdrawal reasons should be clarified and registered on the CRF. Researchers should evaluate, judge, and follow up on adverse events that lead to patient withdrawal. Study medications that have been assigned to withdrawing patients cannot be reassigned to other patients. Subjects who quit cannot be replaced. If a subject withdraws before the end of the study, related assessments should be performed for the subject at the end of the study.

### Sample Size Calculation

This is a superiority trial, and the sample size calculation is based on the primary efficacy endpoint, namely the percentage of change in LDL-C levels from baseline to week 4 after randomization. In order to have 90% power to detect clinically meaningful differences in treatment effects with a one-sided significance level of 5% (α=0.05), a sample size of 193 patients is required for each treatment arm. Assuming that the drop-out rate is 20%, this study aims to enroll approximately 484 patients, with 242 patients in the evolocumab plus statin arm and 242 in the statin-only arm. Therefore, the proposed study is expected to enroll a total of 500 patients with acute phase ACS, with 250 patients in each treatment arm.

The number of patients enrolled from each hospital is based on the bed size of each hospital. The Tianjin Chest Hospital plans to enroll 220 patients, and the remaining seven hospitals each will enroll 40 patients.

### Statistical Analysis

The primary efficacy analysis will be based on the time from randomization assignment to the first occurrence of any component of MACE. Data will be analyzed using intention-to-treat analysis (ITT) and per protocol (PP) to assess the efficacy and safety of evolocumab in combination with moderate-intensity statin therapy. The superiority of evolocumab will be assessed for all efficacy endpoint. Continuous variables with an approximately normal distribution will be reported with mean±standard deviation (mean±SD), and differences between the two treatment arms will be tested using independent sample t-test. For continuous variables that are not normally distributed, the median and interquartile range (IQR) will be given, and the nonparametric Mann–Whiney U test will be conducted to test for differences between treatment arms. Discrete variables will be reported with counts and percentage for each arm, and differences between arms will be examined using Fisher’s exact test. In order to compare MACE differences, Kaplan–Meier survival curve analysis will be performed, and hazard ratio (HR) and 95% CI will be calculated. Log-rank tests will also be performed to evaluate the differences in significance and verify treatment efficacy.

The ITT population will include all randomized patients and the PP population will include all randomized patients who complete the entire study. Subjects with missing data will be excluded from the corresponding analysis. Logistic regression will be used to analyze differences in the central effect sizes.

Results of the proposed study will be analyzed using SAS 9.2 or higher version (SAS Institute, North Carolina, United States) software. All statistical tests will be two-tailed unless otherwise indicated or presented with a 95% confidence interval (CI), and statistical significance will be established as *p*<0.05.

## Discussion

The EMSIACS is the first randomized, multicenter trial to investigate the feasibility, safety and efficacy of evolocumab added to moderate-intensity statin therapy specifically for Chinese patients with acute phase ACS. Patients with ACS are at very high risk of recurrent cardiovascular diseases, namely death from cardiovascular causes, MI, repeated coronary revascularization, ischemic stroke, or unstable angina requiring hospitalization (Schwartz et al., 2014). It has been well established that LDL-C is a modifiable risk factor for cardiovascular events, as reducing LDL-C levels in patients presenting with ASCVD or ACS can effectively decrease the incidence of MACE (Sabatine et al., 2016; Koskinas et al., 2018). Statins have long been used as an effective LDL-C lowering agents. However, despite receiving sustained, high-intensity statin therapy, many ACS patients still cannot achieve the target reduction of LDL-C as recommended by the current guidelines (Fox et al., 2018). As a result, the residual risk of mortality is still high. Moreover, statins may have side effects including muscle pain, liver damage, and increased risk of type 2 diabetes (Bandyopadhyay et al., 2018). As the Chinese population is more vulnerable to hepatic diseases, using high-intensity statin therapy alone may not be suitable for Chinese ACS patients.

Monoclonal antibodies target to PCSK9 are relatively novel lipid-lowering agents that have benefits in reducing the risk of cardiovascular disease (Giugliano et al., 2017; Schwartz et al., 2018; Koskinas et al., 2019). To date, two different anti-PCSK9 antibodies have been approved and used in clinical trials to investigate the efficacy of reducing LDL-C levels in other ethnic populations. In the FOURIER trial (Giugliano et al., 2017), the investigators conducted a multinational study where a total of 25,982 patients with clinically evident vascular diseases were randomized into the evolocumab and the matching placebo groups on a background of high-intensity statin therapy. The authors found that patients achieved progressively lower LDL cholesterol concentrations at 4weeks from baseline had progressively fewer cardiovascular events with no increase in adverse events. Moreover, they demonstrated that lowering LDL-C to well below the recommended level in current guidelines (e.g., <0.5mmol/L) is safe for the highest-risk patients. In the EVOPACS trial (Koskinas et al., 2019), the investigators tested the feasibility, safety, and LDL-C-lowering efficacy of evolocumab on top of atorvastatin in European ACS patients for early reduction of LDL-C levels. The results showed a significant reduction of LDL-C at week 8 in the evolocumab group as compared with the placebo group. Moreover, compared with placebo, evolocumab may further benefit patients as evolocumab significantly reduced other atherosclerotic lipid particles, including total cholesterol, apolipoprotein B, non-HDL-C, triglycerides and lipoproteins(a). In ODYSSEY OUTCOMES trial (Schwartz et al., 2018), another type of anti-PCSK9 antibody, alirocumab, was used. Despite different design, the ODYSSEY OUTCOMES study suggested that the risks of any coronary heart disease event, major coronary heart disease events, any cardiovascular event, and a composite of death from any cause, nonfatal myocardial infarction, or nonfatal ischemic stroke were lower among patients treated with alirocumab than among those who received placebo. The authors concluded that alirocumab added to intensive statin therapy reduces cardiovascular morbidity and mortality after ACS. Compared with these studies, our trial design will use evolocumab added to moderate-intensity statins instead of high-intensity statins and will focus on the Chinese population because using a higher dose of statin can easily induce liver injury and myopathy.

Nevertheless, compared to the results of FOURIER, EVOPACS and ODYSSEY OUTCOMES studies, the evolocumab added to moderate-intensity statin therapy is expected to reduce the LDL-C level considerably in Chinese ACS patients and to achieve the LDL-C goal recommended by the China expert consensus/advice (2020; China Cholesterol Education Program Working Committee et al., 2020). In addition, the dosage of the study drug should be tolerable with our patients. EVOPACS trial has shown that decreasing LDL-C even to 0.8mmol/L (30mg/dl) or lower will not increase the risk of adverse responses such as musculoskeletal pain, liver damage, new-onset diabetes mellitus and neurocognitive impairment (Koskinas et al., 2019). Nevertheless, for the Chinese patients with ACS, the actual feasibility, safety, and efficacy of evolocumab added to moderate-intensity statin therapy should be verified. Moreover, the effects of this therapy on inflammatory responses, liver and kidney functions, platelet functions, and atherosclerotic plaque formation in the study population remain unknown. The proposed EMSIACS trial is designed to address these issues.

## Conclusion

This study is the first to evaluate the efficacy of evolocumab added to moderate-intensity statin therapy in lowering LDL-C specifically for the Chinese patients with acute phase ACS. The outcomes of the EMSIACS study will provide insights into the ability of evolocumab to reduce LDL-C over time in Chinese patients who recently experienced with ACS and who, despite high-dosage statin therapy, fail to achieve LDL-C target levels.

## Data Availability Statement

The original contributions presented in the study are included in the article/supplementary material, further inquiries can be directed to the corresponding author.

## Author Contributions

YL and JG were the principal investigators for managing the protocol and developed the protocol for this project. J-YX and M-DG were responsible for data collection and management. J-YL and P-JL were responsible for epidemiological investigations and laboratory testing. C-PL and ZC performed all statistical analysis. YL, JG, J-YL, and P-JL were involved in the initial draft of the manuscript writing. The manuscript was amended based on comments from all authors. All authors contributed to the article and approved the submitted version.

## Funding

This research project was funded by the Key Project of Scientific and Technological Support Plan of Tianjin in 2020 (No. 20YFZCSY00820).

## Conflict of Interest

The authors declare that the research was conducted in the absence of any commercial or financial relationships that could be construed as a potential conflict of interest.

## Publisher’s Note

All claims expressed in this article are solely those of the authors and do not necessarily represent those of their affiliated organizations, or those of the publisher, the editors and the reviewers. Any product that may be evaluated in this article, or claim that may be made by its manufacturer, is not guaranteed or endorsed by the publisher.
